# Supplementary material for: Numerical Analysis of GNSS Signal Outage Effect on EOPs Solutions Using Tightly Coupled GNSS/IMU Integration: A Simulated Case Study in Sweden
Source: Sensors (Basel). 2023 Jul 13;23(14):6361. doi: 10.3390/s23146361 (PMC10383582; doi:10.3390/s23146361)
Supplement: Supplementary file 1 [file sensors-23-06361-s001.zip › sensors-2390240-supplementary.pdf]

Supplementary Materials for

Numerical analysis of GNSS signal outage effect on EOPs solutions using tightly coupled GNSS/IMU integration: A simulated case study in Sweden

Arash Jouybari [1], Mohammad Bagherbandi [1,2] and Faramarz Nilfouroushan [1,3]

[1] Faculty of Engineering and Sustainable Development, University of Gävle, SE-80176 Gävle, Sweden; arash.jouybari@hig.se (A.J.); mohammad.bagherbandi@hig.se (M.B.); faramarz.nilfouroushan@hig.se (F.N.)
[2] Division of Surveying-Geodesy, Land Law and Real Estate Planning, Royal Institute of Technology (KTH), SE-10044 Stockholm, Sweden
[3] Department of Geodetic Infrastructure, Geodata Division, Lantmäteriet, SE-80182 Gävle, Sweden

This file includes:

Figure S1. A basic schematic of smoothing sequence and post-processing modes.

Figure S2. Attitude uncertainties in tightly- and loosely-coupled network-based PPK with smoothing algorithm for 180 seconds GNSS signal outage.

Figure S3. Position uncertainties in tightly- and loosely-coupled network-based PPK with forward KF for 180 seconds GNSS signal outage.

Figure S4. Attitude uncertainties in tightly- and loosely-coupled network-based PPK with forward KF for 180 seconds GNSS signal outage.

Figure S5. East uncertainties in single-based PPK with forward KF, network-based PPK with forward KF, single-based PPK with smooth processing, and network-based PPK smooth processing modes.

Figure S6. Height uncertainties in single-based PPK with forward KF, network-based PPK with forward KF, single-based PPK with smooth processing, and network-based PPK smooth processing modes.

Figure S7. Roll uncertainties in single-based PPK with forward KF, network-based PPK with forward KF, single-based PPK with smooth processing, and network-based PPK smooth processing modes.

Figure S8. Pitch uncertainties in single-based PPK with forward KF, network-based PPK with forward KF, single-based PPK with smooth processing, and network-based PPK smooth processing modes.

Figure S9. Heading uncertainties in single-based PPK with forward KF, network-based PPK with forward KF, single-based PPK with smooth processing, and network-based PPK smooth processing modes.

Figure S10. 2D position (north and east) uncertainties using network-based PPK with forward KF processing and assuming different GNSS outage durations.

Figure S11. Height uncertainties using network-based PPK with forward KF processing and assuming different GNSS outage durations.

Figure S12. Uncertainties of the roll using network-based PPK with forward KF processing and assuming different GNSS signal outages.

Figure S13. Uncertainties of the pitch using network-based PPK with forward KF processing and assuming different GNSS signal outages.

Figure S14. Uncertainties of heading using network-based PPK with forward KF processing and assuming different GNSS outage durations.

Table. S1. Statistical information of EOPs uncertainties in single-based PPK with forward KF (SF), network-based PPK with forward KF (NF), single-based PPK with smooth processing (SS), and network-base with smooth processing (NS).

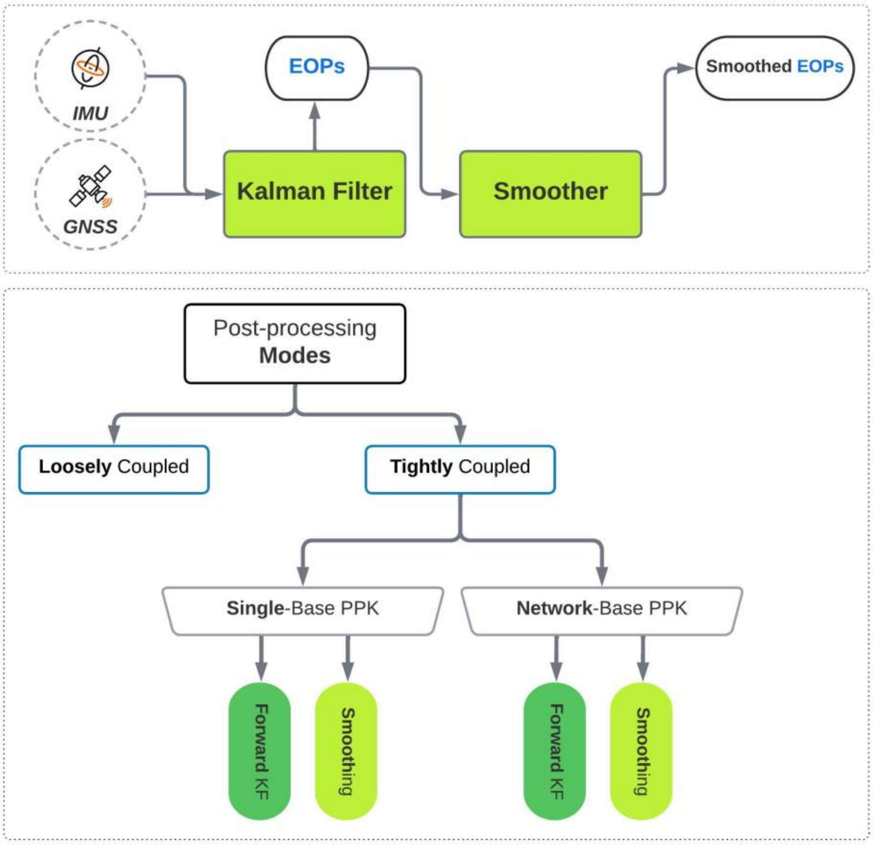

Figure S1. A basic schematic of smoothing sequence (up) and post-processing modes (down).
Note: EOPs in the upper flowchart are the results of the forward Kalman filter, which will be smoothed with the smoother algorithm in the next step.

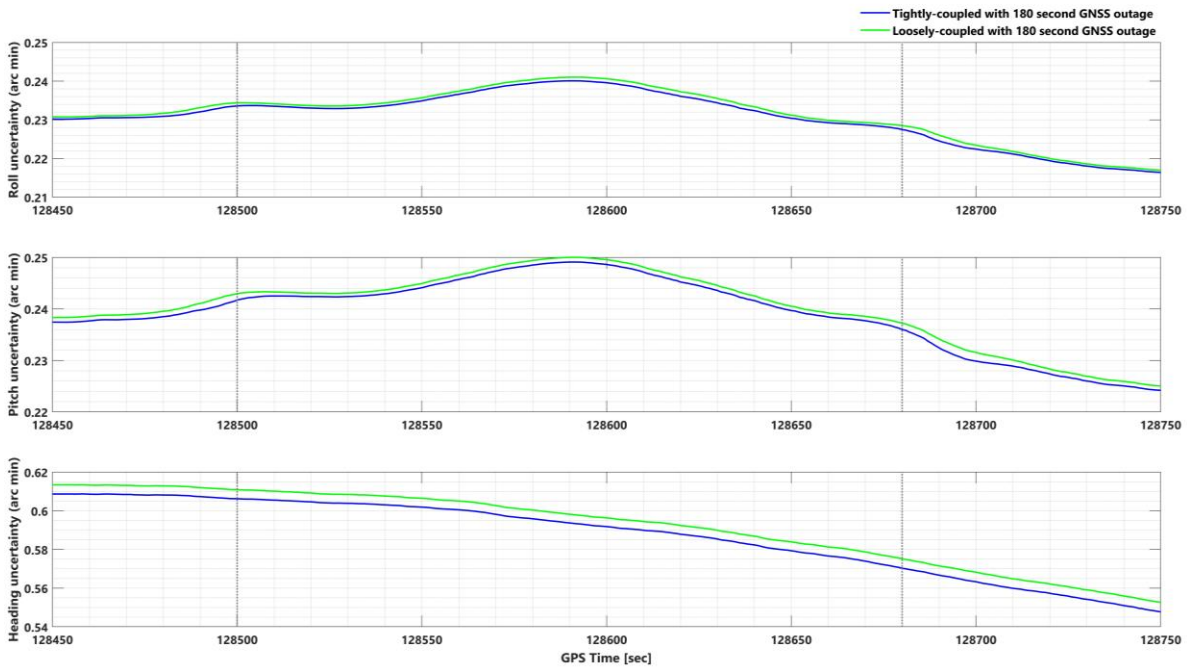

Figure S2. Attitude uncertainties in tightly- and loosely-coupled network-based PPK with smoothing algorithm for 180 seconds GNSS signal outage.

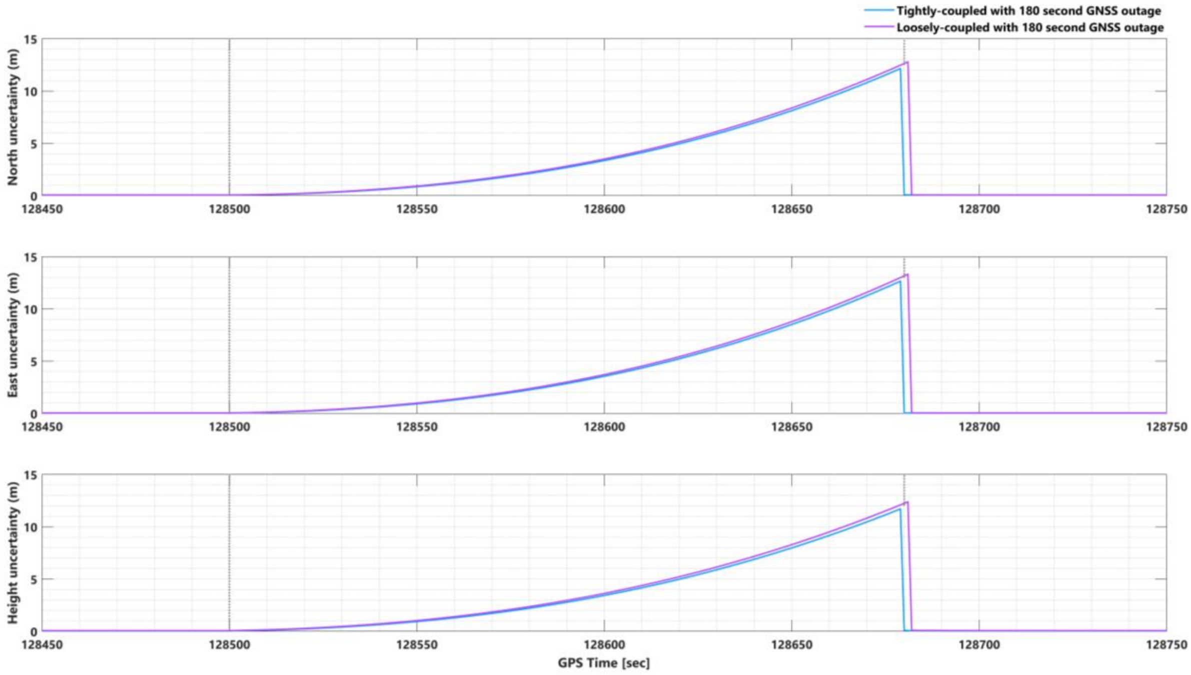

Figure S3. Position uncertainties in tightly- and loosely-coupled network-based PPK with forward KF for 180 seconds GNSS signal outage.

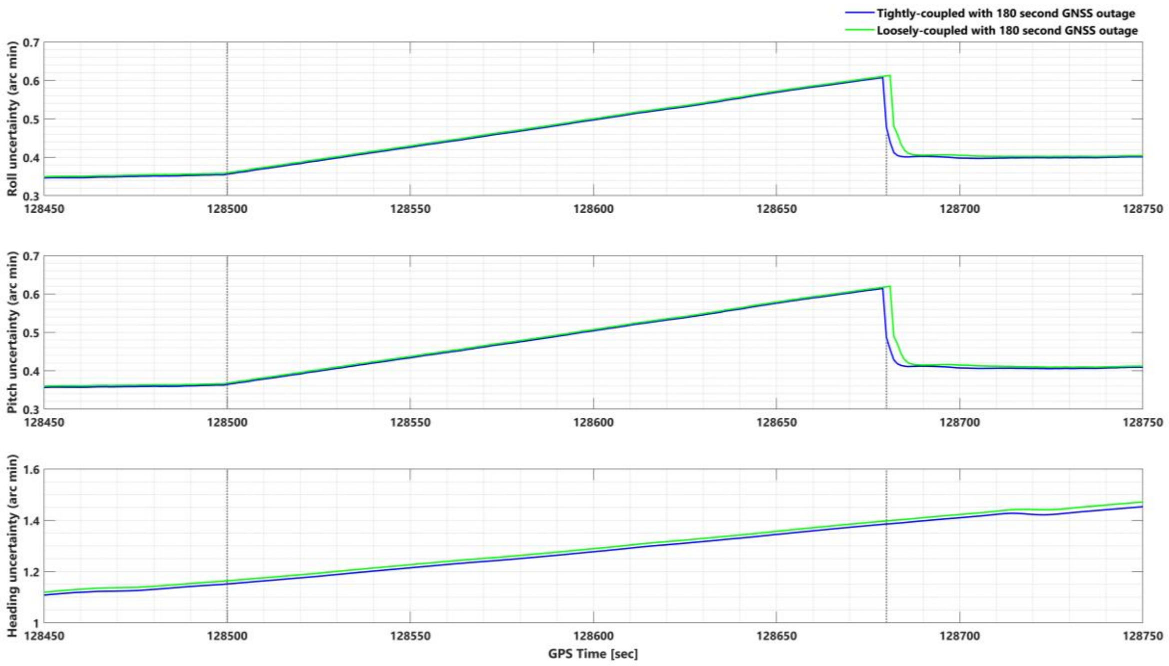

Figure S4. Attitude uncertainties in tightly- and loosely-coupled network-based PPK with forward KF for 180 seconds GNSS signal outage.

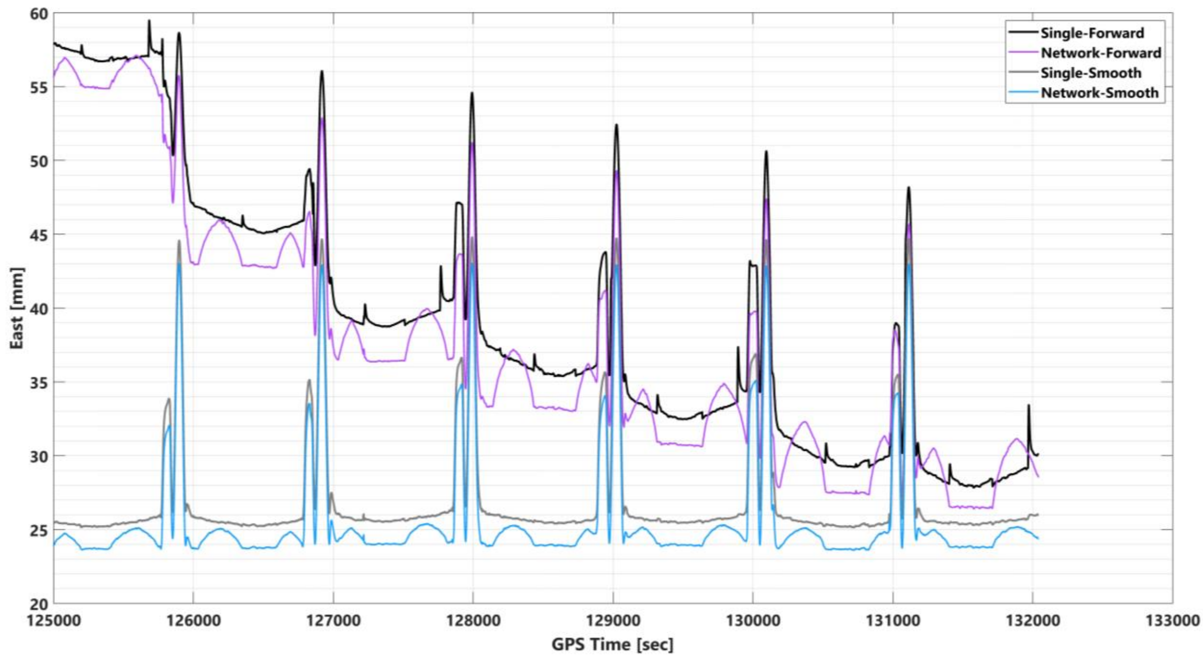

Figure S5. East uncertainties in single-based PPK with forward KF, network-based PPK with forward KF, single-based PPK with smooth processing, and network-based PPK smooth processing modes.

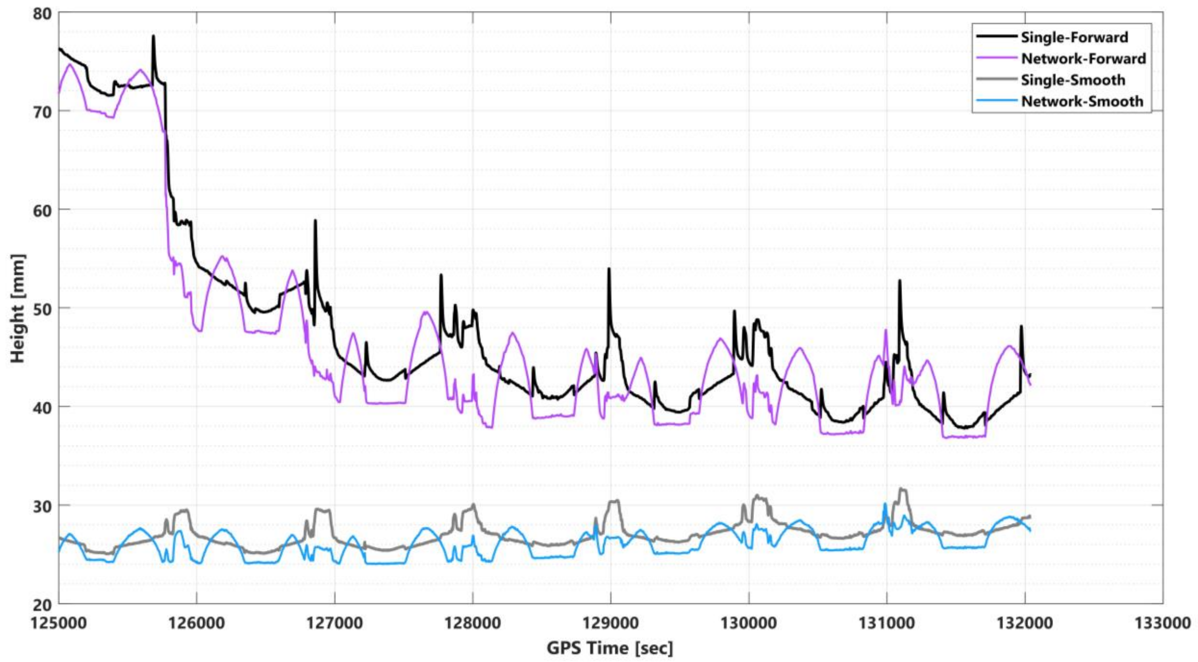

Figure S6. Height uncertainties in single-based PPK with forward KF, network-based PPK with forward KF, single-based PPK with smooth processing, and network-based PPK smooth processing modes.

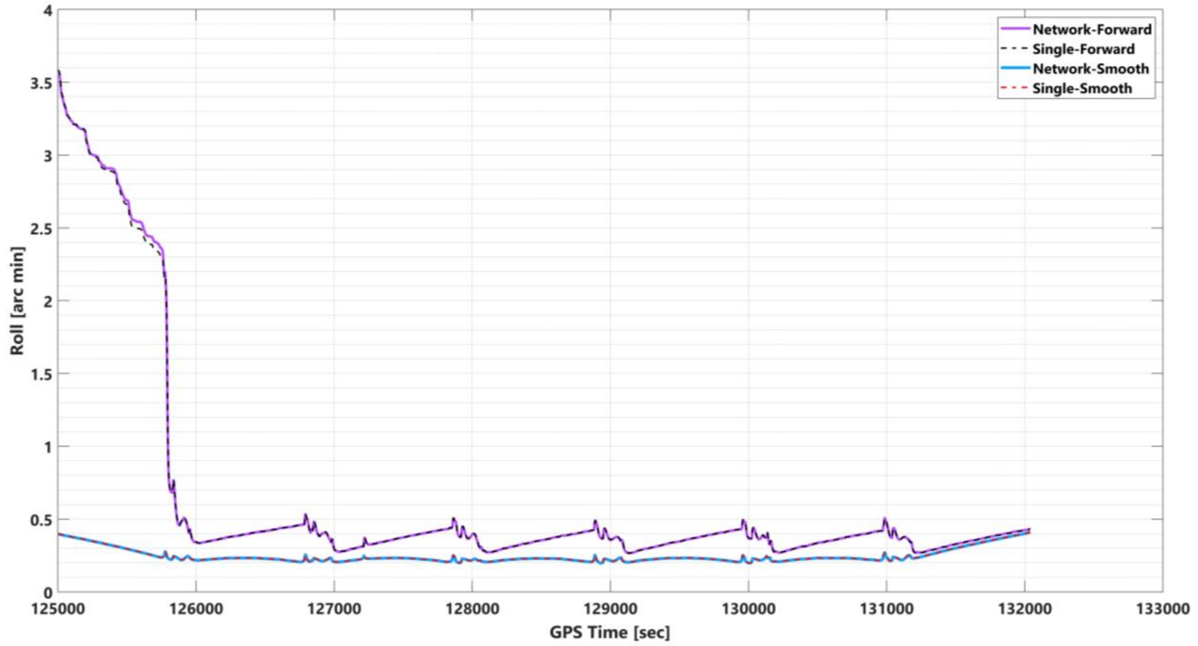

Figure S7. Roll uncertainties in single-based PPK with forward KF, network-based PPK with forward KF, single-based PPK with smooth processing, and network-based PPK smooth processing modes.

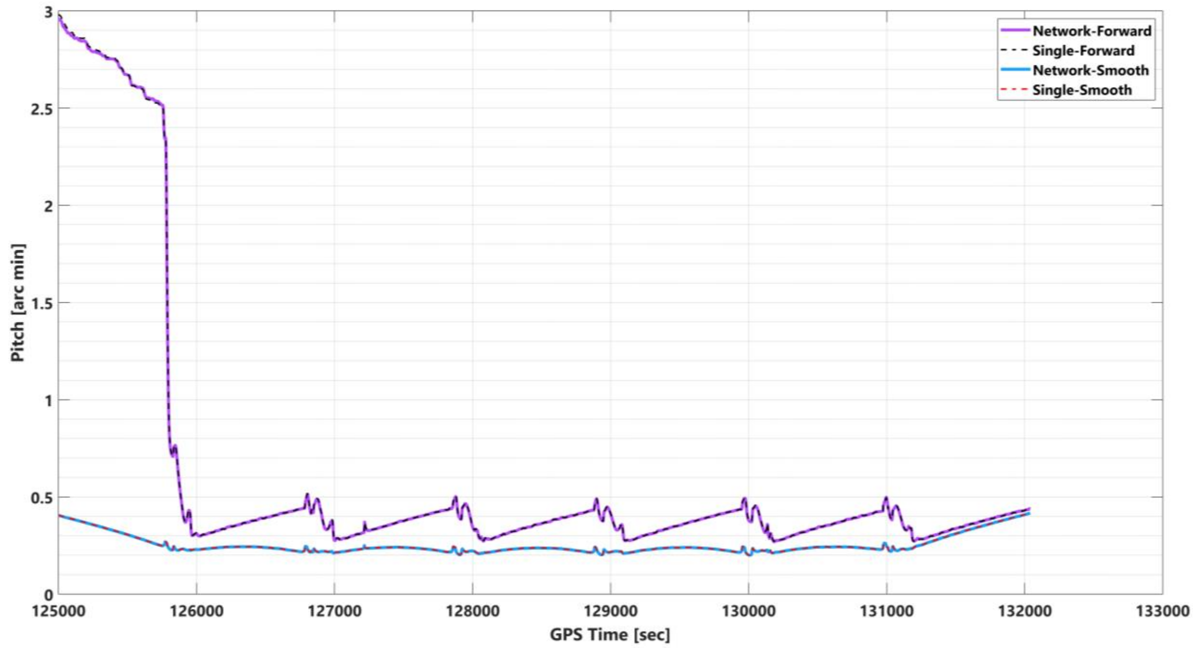

Figure S8. Pitch uncertainties in single-based PPK with forward KF, network-based PPK with forward KF, single-based PPK with smooth processing, and network-based PPK smooth processing modes.

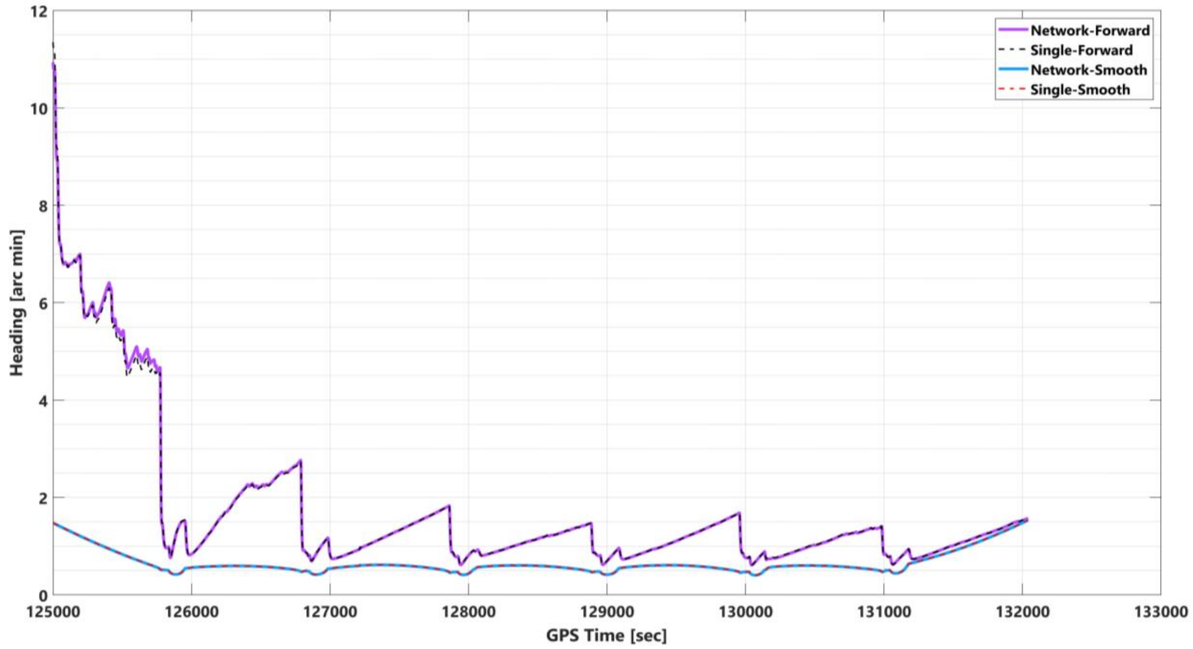

Figure S9. Heading uncertainties in single-based PPK with forward KF, network-based PPK with forward KF, single-based PPK with smooth processing, and network-based PPK smooth processing modes.

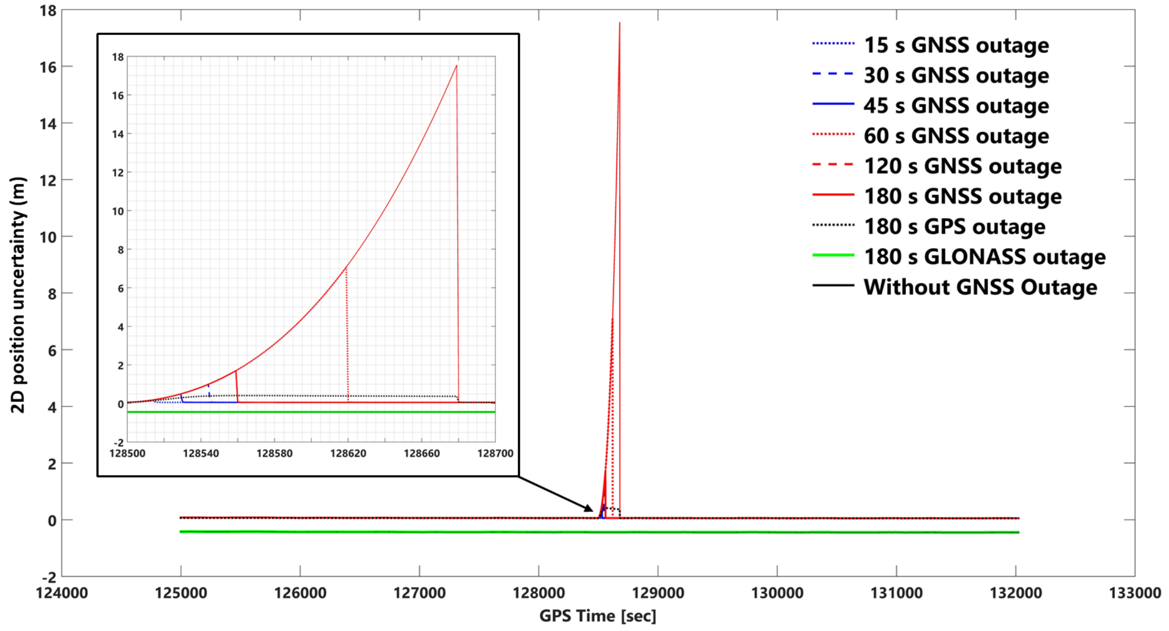

Figure S10. 2D position (north and east) uncertainties using network-based PPK with **forward KF** processing and assuming different GNSS outage durations.

Note: for better illustration:

- The inset figure shows the focused zoom of the main plot during the GNSS signal outage.
- We set – 0.05 meter shift for 180 seconds GLONASS outage and without GNSS outage plots for both inset and whole mission plots.

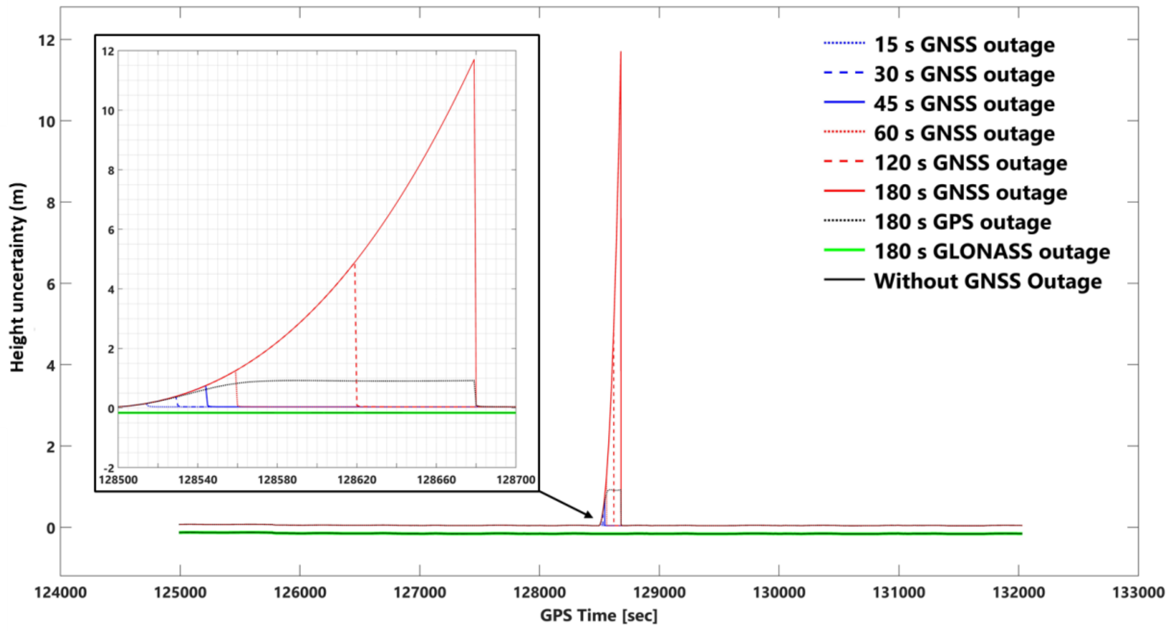

Figure S11. Height uncertainties using network-based PPK with **forward KF** processing and assuming different GNSS outage durations.

Note: for better illustration:

- The inset figure shows the focused zoom of the main plot during the GNSS signal outage.
- We set – 0.2 meter shift for 180 seconds GLONASS outage and without GNSS outage plots for both inset and whole mission plots.

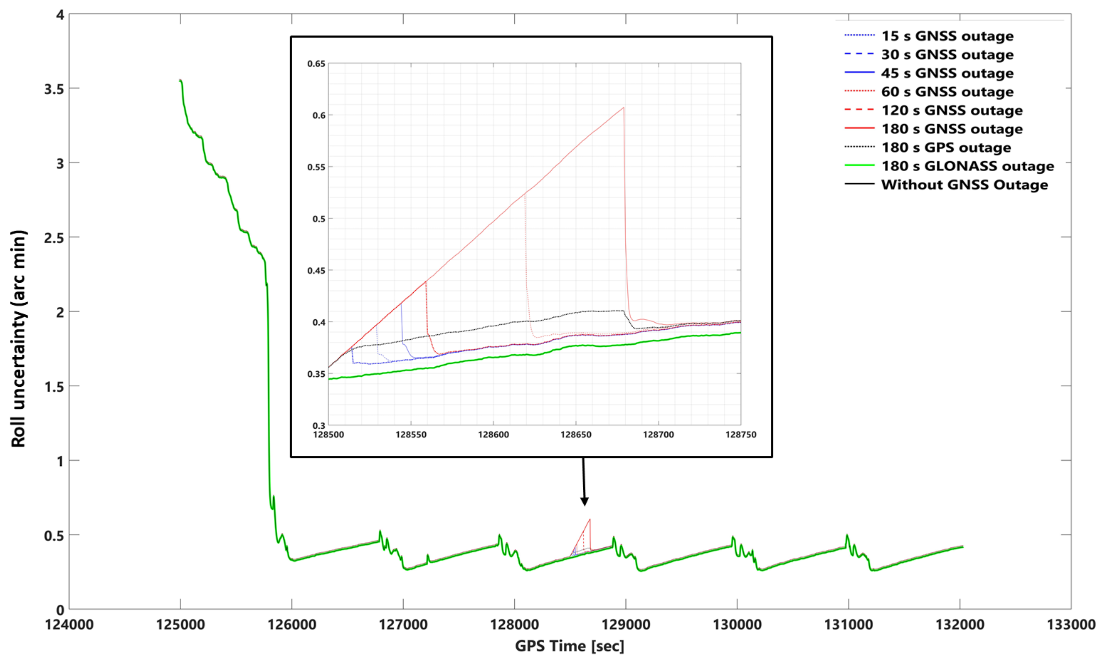

Figure S12. Uncertainties of the **roll** using network-based PPK with **forward KF** processing and assuming different GNSS signal outages.

Note: for better illustration:

- The inset figure shows the focused zoom of the main plot during the GNSS signal outage.
- We set – 0.01 arc minute shift for 180 seconds GLONASS outage and without GNSS outage plots for both inset and whole mission plots.

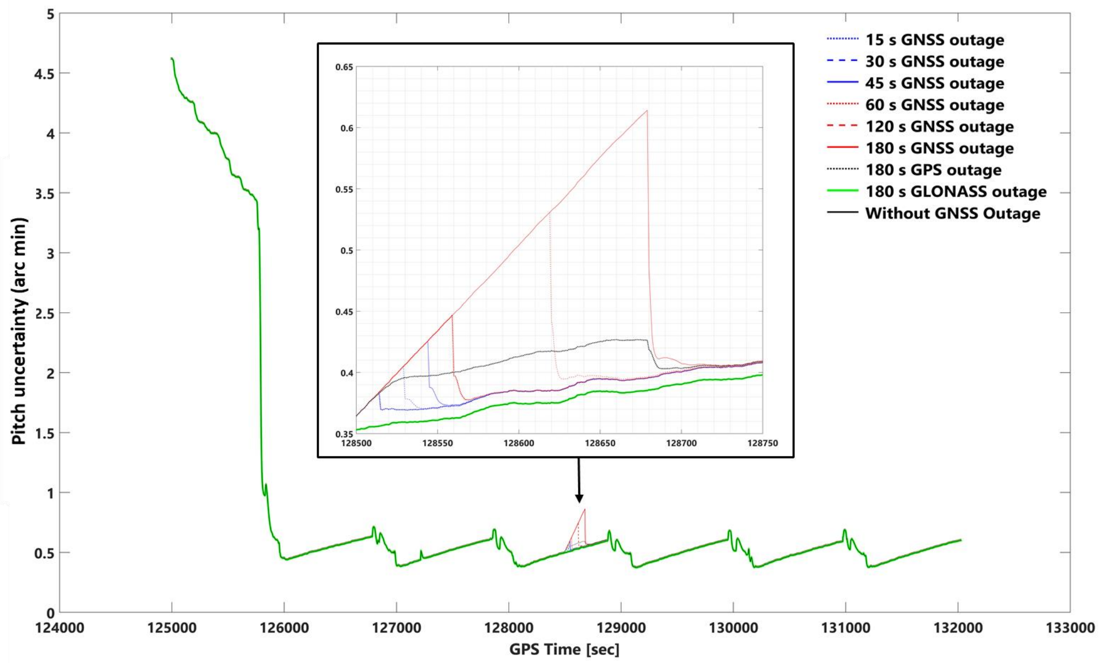

Figure S13. Uncertainties of the **pitch** using network-based PPK with **forward KF** processing and assuming different GNSS signal outages.

Note: for better illustration:

- The inset figure shows the focused zoom of the main plot during the GNSS signal outage.
- We set – 0.01 arc minute shift for 180 seconds GLONASS outage and without GNSS outage plots for both inset and whole mission plots.

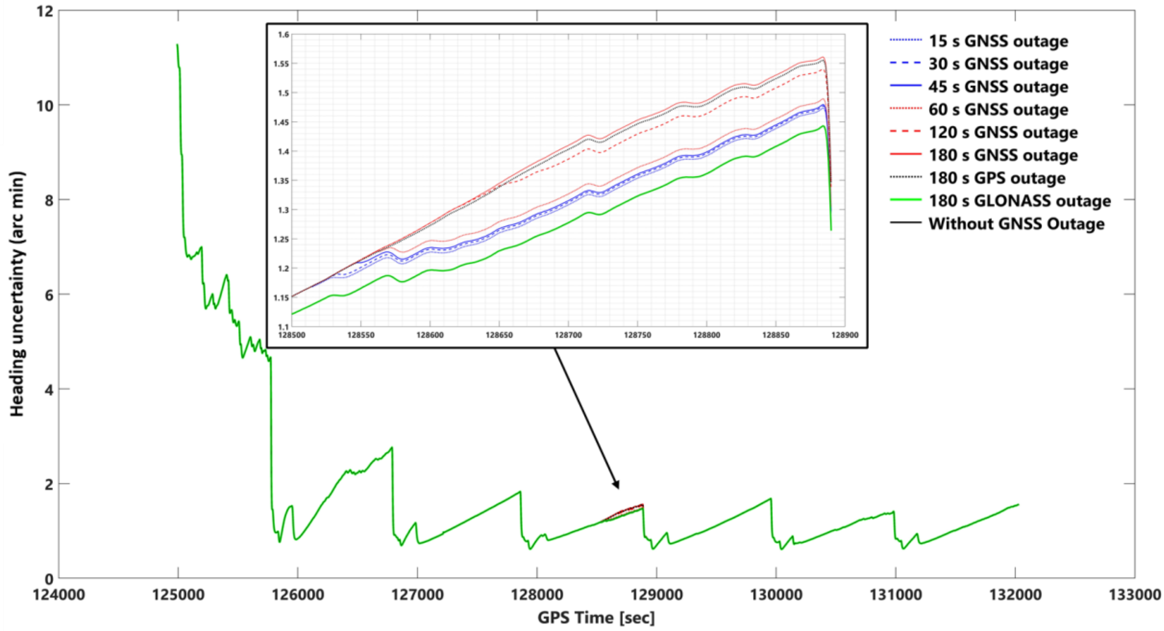

Figure S14. Uncertainties of **heading** using network-based PPK with **forward KF** processing and assuming different GNSS outage durations.

Note: for better illustration:

- The inset figure shows the focused zoom of the main plot during the GNSS signal outage.
- We set – 0.03 arc minute shift for 180 seconds GLONASS outage and without GNSS outage plots for both inset and whole mission plot

Table S1. Statistical information of EOPs uncertainties in single-based PPK with forward KF (SF), network-based PPK with forward KF (NF), single-based PPK with smooth processing (SS), and network-base with smooth processing (NS).

EOPs	Processing Modes	Units	Max	Mean	Min	STD
North	SF	mm	63	52	32	5.2
	NF		60	51	29	5.2
	SS		45	43	27	3
	NS		44	42	24	3.2
East	SF	mm	59	39	28	9.1
	NF		57	38	26	8.7
	SS		45	27	25	3.9
	NS		43	26	24	3.7
Height	SF	mm	78	48	38	10
	NF		75	46	37	9.9
	SS		32	27	25	1.3
	NS		30	26	24	1.3
Roll	SF	arc min	3.6	0.65	0.27	0.78
	NF		3.6	0.65	0.27	0.79
	SS		0.41	0.25	0.2	0.048
	NS		0.41	0.25	0.2	0.048
Pitch	SF	arc min	3	0.64	0.27	0.74
	NF		3	0.63	0.27	0.74
	SS		0.42	0.25	0.2	0.049
	NS		0.42	0.25	0.2	0.049
Heading	SF	arc min	11	1.7	0.62	1.6
	NF		11	1.7	0.61	1.6
	SS		1.5	0.66	0.41	0.24
	NS		1.5	0.66	0.4	0.24
